# Single-Cell Transcriptomics of Proliferative Phase Endometrium: Systems Analysis of Cell–Cell Communication Network Using CellChat

**DOI:** 10.3389/fcell.2022.919731

**Published:** 2022-07-22

**Authors:** Zishui Fang, Yao Tian, Cong Sui, Yaxin Guo, Xinyao Hu, Youhua Lai, Zhiqi Liao, Jie Li, Guihai Feng, Lei Jin, Kun Qian

**Affiliations:** ^1^ Reproductive Medicine Center, Tongji Hospital, Tongji Medical College, Huazhong University of Science and Technology, Wuhan, China; ^2^ State Key Laboratory of Stem Cell and Reproductive Biology, Institute of Zoology, Chinese Academy of Sciences, Beijing, China

**Keywords:** single-cell sequencing, endometrium, proliferation, angiogenesis, cell communication network

## Abstract

The endometrium thickness increases by which endometrial angiogenesis occurs in parallel with the rapid growth of endometrium during the proliferative phase, which is orchestrated by complex cell–cell interactions and cytokine networks. However, the intercellular communication has not been fully delineated. In the present work, we studied the cell–cell interactome among cells of human proliferative phase endometrium using single-cell transcriptomics. The transcriptomes of 33,240 primary endometrial cells were profiled at single-cell resolution. CellChat was used to infer the cell–cell interactome by assessing the gene expression of receptor–ligand pairs across cell types. In total, nine cell types and 88 functionally related signaling pathways were found. Among them, growth factors and angiogenic factor signaling pathways, including EGF, FGF, IGF, PDGF, TGFb, VEGF, ANGPT, and ANGPTL that are highly associated with endometrial growth, were further analyzed and verified. The results showed that stromal cells and proliferating stromal cells represented cell–cell interaction hubs with a large number of EGF, PDGF incoming signals, and FGF outgoing signals. Endothelial cells exhibited cell–cell interaction hubs with a plenty of VEGF, TGFb incoming signals, and ANGPT outgoing signals. Unciliated epithelial cells, ciliated epithelial cells, and macrophages exhibited cell–cell interaction hubs with substantial EGF outgoing signals. Ciliated epithelial cells represented cell–cell interaction hubs with a large number of IGF and TGFb incoming signals. Smooth muscle cells represented lots of PDGF incoming signals and ANGPT and ANGPTL outgoing signals. This study deconvoluted complex intercellular communications at the single-cell level and predicted meaningful biological discoveries, which deepened the understanding of communications among endometrial cells.

## Introduction

Repeated shedding of the endometrium necessitates complete repair and regeneration of the denuded surface. During the proliferative phase, there is a rapid growth of the functional layer of the endometrium, necessitating angiogenesis to maintain perfusion of new tissue (Girling and Rogers, 2005). Angiogenesis is a biological process that involved endothelial cell proliferation, migration, and the formation of new vessels from pre-existing capillaries (Thompson et al., 2000; Dragneva et al., 2013), while another set is involved in the uterine gland and stroma remodeling (Ji et al., 2013; Maltepe and Fisher, 2015). Importantly, 17β-estradiol (E2) plays a role in the reconstruction of new vascular networks and rapid vessel growth in the endometrium. In addition to hormonal signals, the development of the endometrium also requires a considerable level of negotiation between cells of endometrium throughout ligands and receptors, especially cytokines and growth factors.

Cell types with different biological functions would show distinct profiles of ligands and receptors, as these enable the cell-type-specific interactions. Therefore, the receptor/ligand profile may serve as a robust biological characterization of the cell types. Recent literature has revealed that ligand–receptor pairs were upregulated, respectively, in stromal fibroblasts and lymphocytes, for example, IL15 and IL2RB, IL2RG, MHC class I genes, and NKR in decidualized endometrium, suggesting a direct interplay between the two cell types (Wang et al., 2020). Furthermore, Vento-Tormo et al. used CellPhoneDB to identify the expression of cytokines and chemokines in decidual natural killer cells (dNKs) and to predict their interactions with other cells at the maternal–fetal interface (Vento-Tormo et al., 2018). Suryawanshi et al. visualized average expression levels of most abundant ligands and their cognate receptors for each cell type’s ligand–receptor pair of the human first-trimester placenta and decidua (Suryawanshi et al., 2018). Though much has been known about the ligands and receptors of decidualized endometrium, to our knowledge, no information is available about the interconnection among endometrial cell types during the proliferative phase.

CellChat, a tool that is able to quantitatively infer, visualize, and analyze intercellular communication networks, can identify key features of intercellular communications within a given single-cell RNA sequencing (scRNA-seq) dataset and predict putative functions for poorly understood signaling pathways. The successful performance of CellChat lies in utilizing a mass action–based model to integrate all known molecular interactions, including the core interaction between the ligands and the receptors with multi-subunit structure and additional modulation by cofactors. CellChat can predict major signaling inputs and outputs for cells and how those cells and signals coordinate for functions using network analysis and pattern recognition approaches. CellChat also can provide several visualization outputs to facilitate intuitive user-guided data interpretation (Jin et al., 2021).

Endometrial architecture significantly changed under the regulation of hormones across the menstrual cycle. However, a rather high degree of variability between individuals in the same menstrual cycle phase makes it inaccurate to interpret data by combining results from different studies. This may be due to differences in hormone levels at the time of sampling. To reduce these problems, hormonally controlled cycles were adopted to reduce interference of the individual heterogeneity in our study.

Here, we implemented scRNA-seq to obtain an unbiased and comprehensive visualization of the endometrial cells during the proliferative phase. Our study depicted a cell–cell panoramic interactome landscape of endometrial cells using CellChat, which will facilitate a better understanding of the regeneration of the endometrium.

## Materials and Methods

### Participants and Materials

The participants recruited were patients who performed *in vitro* fertilization (IVF) due to male factors (e.g., oligospermia, asthenospermia, and azoospermia). The inclusion criteria were age between 18 and 32 years, ordinary menstrual bleedings, cessation of medical treatment, removal of intra-uterine device or cessation of hormonal contraception, body mass index (BMI) between 20 and 24, and no history of former pathology concerning the endometrium or myometrium. The exclusion criteria included any contra-indication to estrogen supplementation (e.g., prior thrombosis, prior or current hormone-sensitive malignancy, and porphyria) and anatomical uterine anomalies. Clinical tests were performed prior to enrollment, including imaging tests, blood routine, hepatitis B and hepatitis C virus immunology, *treponema* syphilis antibody, HIV antibody, coagulation function, liver and kidney function, vaginal secretion *mycoplasma*, *chlamydia*, gonococcal culture, endometrial biopsy, shedding cell examination, electrocardiogram, and urine routine to ensure that the patient was healthy. The tissue samples used for this study were obtained with written informed consent from all participants. The current study was approved by the ethical committee of Tongji Hospital (No. TJ-IRB20210909). Unless otherwise stated, all reagents were purchased from Sigma-Aldrich, and all immunofluorescence antibodies were obtained from Abcam.

### Hormonally Controlled Cycles

In hormonally controlled cycles, estradiol valerate (Shire Pharmaceuticals, Dublin, Ireland, United Kingdom) was taken orally at 4 mg/day from day 2 to day 5, at 6 mg/day from day 6 to day 9, and at 8 mg/day from day 10 onward. The estrogen level in the blood at this time is stable between 200 and 300 pg/ml. When the endometrial thickness reached ≥ 8 mm (Lewin et al., 2002; Hawkins Bressler et al., 2021), participants were included in the study, and then an endometrial tissue biopsy was performed. If the endometrium remained <8 mm, the hormonally controlled cycle was canceled. For endometrium biopsy, we used a small scraping spoon to probe the depth of the uterine cavity and scratch the front and rear wall, the side wall, and the bottom of the cavity until a rough feeling.

### Construct Library and Sequencing

For droplet-based scRNA-seq, the tissues of the endometrium (*N* = 3) were pooled. Cells were counted, and ∼13,000 cells were loaded per channel onto a chromium controller (10× Genomics) for the generation of gel bead-in-emulsions. Sequencing libraries were prepared using Single Cell 3′ Reagent Kits v3 (10× Genomics) and then converted using the MGIEasy Universal Library Conversion Kit (BGI) before sequencing on a MGISEQ-2000 instrument (BGI). For the BGI FASTQ files to be made compatible with the “cellranger count” pipeline from Cell Ranger version 3.0.2 (10× Genomics), the file names and the FASTQ headers were reformatted using the code from https://github.com/IMB-Computational-Genomics-Lab/BGIvsIllumina_scRNASeq. Data were processed using Homo_sapiens GCF_000001405.39_GRCh38.p13 as a reference.

### Analysis of Single-Cell Transcriptomes

Sequencing data were first preprocessed through the Cell Ranger pipeline (10× Genomics, Cellranger count v5) with default parameters, aligned to GRCh38 (v3.0.0), and the resulting matrix files were used for subsequent bioinformatics analyses. Seurat (version 3.1.5 and R version 4.0.2, R) was utilized for downstream analysis. Cells with at least 600 detected genes and mitochondrial percentage <15% were retained, and the data were normalized to transcript copies per 10,000 and log-normalized to reduce sequencing depth variability. For visualization and clustering, manifolds were calculated using UMAP methods (RunUMAP, Seurat) on 20 precomputed principal components. Clusters were identified by calculating a shared-neighbor graph and then defined (FindClusters, Seurat) with a resolution of 0.2. Identification of differentially expressed genes between clusters was carried out using the default Wilcoxon rank sum test (Seurat). For comparison with published data, SingleR was used with published data as a reference object to project the cell type onto the data.

### Cell–Cell Communication Analysis

For the inference and analysis of cell–cell communication, we used CellChat (1.1.0), a public repository of ligands, receptors, cofactors, and their interactions. The versatile and easy-to-use toolkit CellChat and a web-based Explorer (http://www.cellchat.org/) help discover novel intercellular communications and build cell–cell communication atlases. For the cell–interaction analysis, the expression levels were calculated relative to the total read mapping to the same set of coding genes in all transcriptomes. The expression values were averaged within each single-cell cluster/cell sample.

### TSA-Multilabel Tissue Immunofluorescence Staining

Formalin-fixed and paraffin-embedded endometrial tissues were cut into 4-μm sections. Antigen retrieval was performed by EDTA buffer (pH 8.0) for 15 min after deparaffinization and rehydration. For immunofluorescence staining, tissue sections were blocked with 3% H_2_O_2_ for 25 min to remove endogenous peroxidase. Then, they were blocked with 5% serum for 30 min at room temperature (RT), incubated overnight at 4°C with primary antibody, and treated with horseradish peroxidase (HRP)–conjugated secondary antibodies for 50 min at RT. Subsequently, CY3-TSA was added and incubated at RT for 10 min in the dark. The tissue sections were repaired with EDTA antigen repair buffer (PH 8.0) in a microwave oven with medium fire for 8 min, ceasefire for 8 min, and low fire for 7 min. After that, the second primary antibody was added and incubated overnight at 4°C. Then, the corresponding HRP-conjugated secondary antibodies were added and incubated for 50 min at RT in the dark. Add FITC-TSA for 10 min at room temperature protected from light. Tissue section antigens were repaired as mentioned before. Then, the third primary antibody was added and incubated overnight at 4°C. The CY5-labeled fluorescent secondary antibody was added and incubated for 50 min at RT in the dark. Nuclei were stained with DAPI for 10 min at RT in the dark. Slices were sealed with anti-fluorescence quenched tablets. The sections were placed under a scanner to collect the images.

## Results

### Single-Cell Transcriptional Profiling of Endometrial Cells

**FIGURE 1:**
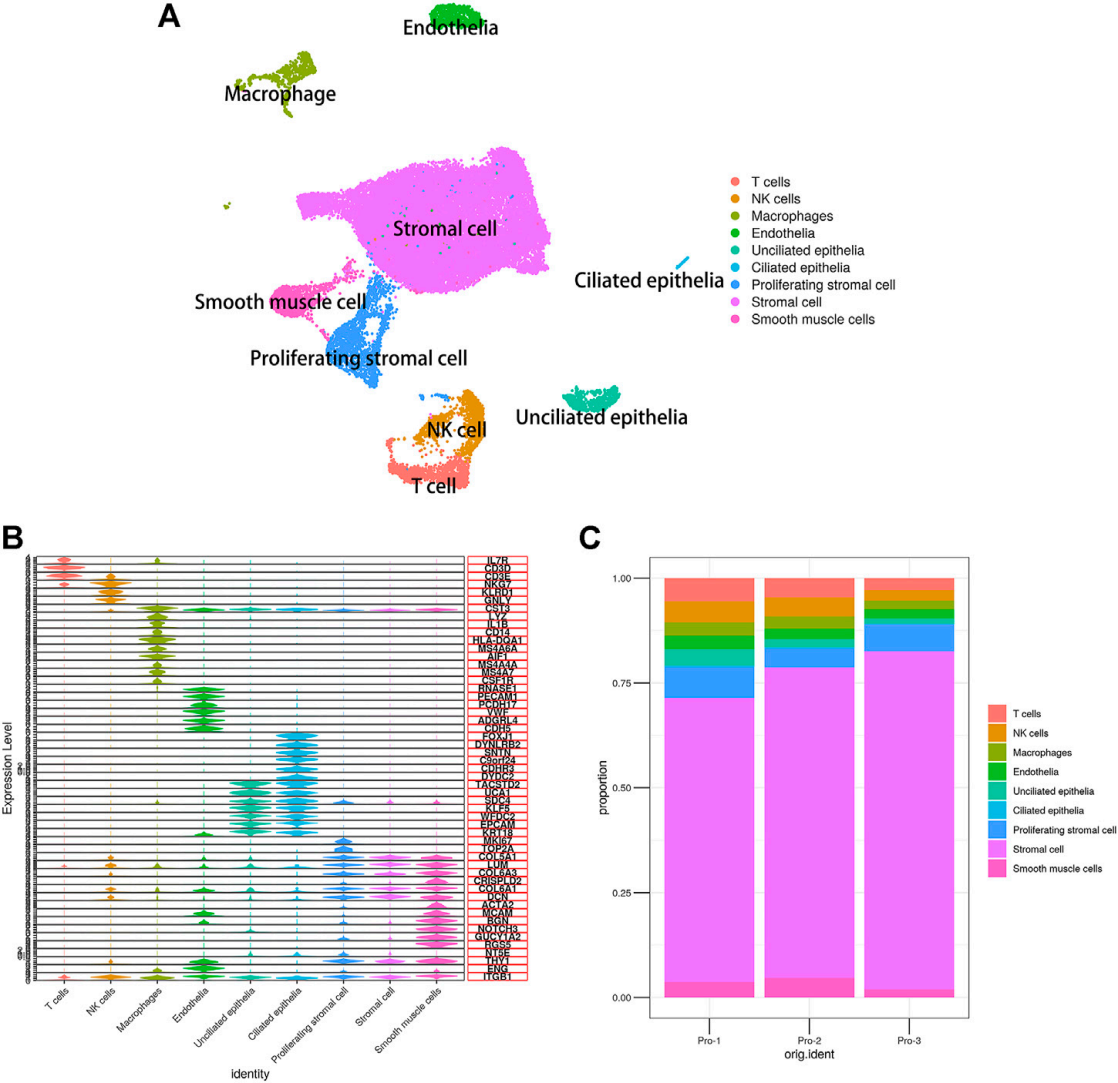
Single-cell expression atlas of human proliferating phase endometrium. **(A)** Cell type assignment following UMAP-based visualization of expression differences for 33,240 single cells from three healthy human proliferating phase endometrium samples using established lineage markers. **(B)** Violin plots showing the expression of known lineage markers and coexpressed lineage-specific genes. **(C)** Proportion of cell types of three healthy human proliferating phase endometrium samples.

To characterize the features of endometrial cells, we performed droplet-based scRNA-seq (10× Genomics) to study the transcriptomic profiles of endometrial cells from three healthy donors. All high-quality cells were integrated into an unbatched and comparable dataset and subjected to principal component analysis after correction for read depth and mitochondrial read counts. Using graph-based clustering of uniform manifold approximation and projection (UMAP), we analyzed the distribution of the endometrial cells. Based on the expression of canonical lineage markers and other genes specifically upregulated in each cluster as well as previous studies (Suryawanshi et al., 2018; Vento-Tormo et al., 2018; Fitzgerald et al., 2019; Lucas et al., 2020; Wang et al., 2020; Lv et al., 2022), we annotated the cell types. As such, we clearly defined the composition of cell populations in the endometrium at the proliferative phase and identified a total of nine cell clusters, namely, stromal cells, endothelial cells, smooth muscle cells (also annotated as pericytes), ciliated epithelia, unciliated epithelia, immune cells (T cells, NK cells, and macrophages), and a discrete but transcriptionally distinct proliferating stromal subpopulation, as shown in Figure 1A. Also, the known lineage markers and co-expressed lineage-specific genes are shown in Figure 1B. The proportion of cell types of three healthy human proliferative phase endometrium samples is shown in Figure 1C.

### Signaling Pathways

**FIGURE 2:**
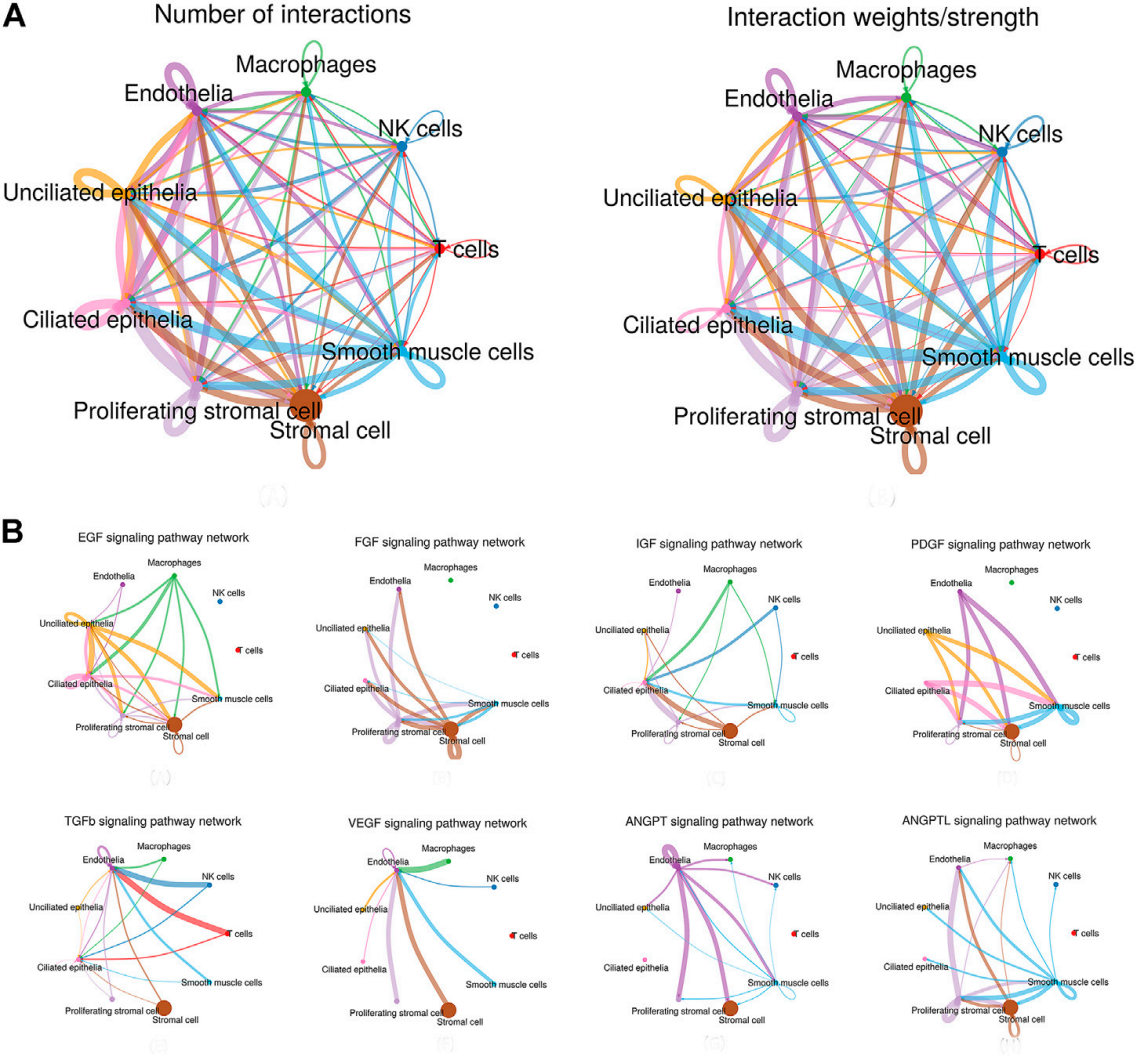
Interaction plot of endometrial cells and intercellular communication networks for spatially colocalized endometrial cell populations. **(A)** Interaction net count plot of endometrial cells. The interaction weight plot of endometrial cells. The thicker the line represented, the more the number of interactions, and the stronger the interaction weights/strength between the two cell types. **(B)** Intercellular communication networks for spatially colocalized endometrial cell populations. The circle plot showed the inferred intercellular communication network for proliferation and angiogenesis-related signaling pathways. The thicker the line represented, the more the number of interactions, and the stronger the interaction weights/strength between the two cell types.

Through manifold learning and quantitative contrasts, CellChat identified differentially over-expressed ligands and receptors for each cell group. In total, 3,917 significant ligand–receptor pairs were detected, which were further categorized into 88 signaling pathways. The results are listed in Table 1. Significant interactions were identified on the basis of a statistical test that randomly permutes the group labels of cells and then recalculates the interaction probability, and the signaling pathways are listed in Supplementary Sheet S1 and the ligand–receptor pairs are listed in Supplementary Sheet S2. In addition, the net counts and the interaction weights are also calculated respectively (Supplementary Sheets S3, S4). Interestingly, according to the results of our analysis, the signaling pathways of nonimmune cells were preponderance than those of immune cells, both in interaction net number and in interaction weight/strength, as shown in Figure 2A. In addition, the ligand–receptor pair types of ECM-receptor pathway contain a large number of ECM–integrin receptor pairs in our study. For example, stromal cells expressed a large amount of ECM and formed ligand–receptor pairs with other cell types, such as COL1A1-(ITGA1 + ITGB1), FN1-(ITGA3 + ITGB1), and LAMA4-(ITGA1 + ITGB1), which explained the prevalence of stromal-based interactions. Furthermore, growth factors and angiogenic factors signaling pathways, including EGF, FGF, IGF, PDGF, TGFb, VEGF, ANGPT, and ANGPTL that are highly associated with endometrial growth, were further analyzed and confirmed.

**TABLE 1:** Type and number of ligand–receptor pairs.

Communication mode	The number of pathways	The number of L–R pair types	The number of L–R pairs
Cell–cell contact	40	102	772
ECM-receptor	7	169	2,140
Secreted signaling	41	147	1,005

Note: L–R, ligand–receptor.

### Epidermal Growth Factor Signaling Pathway

**FIGURE 3:**
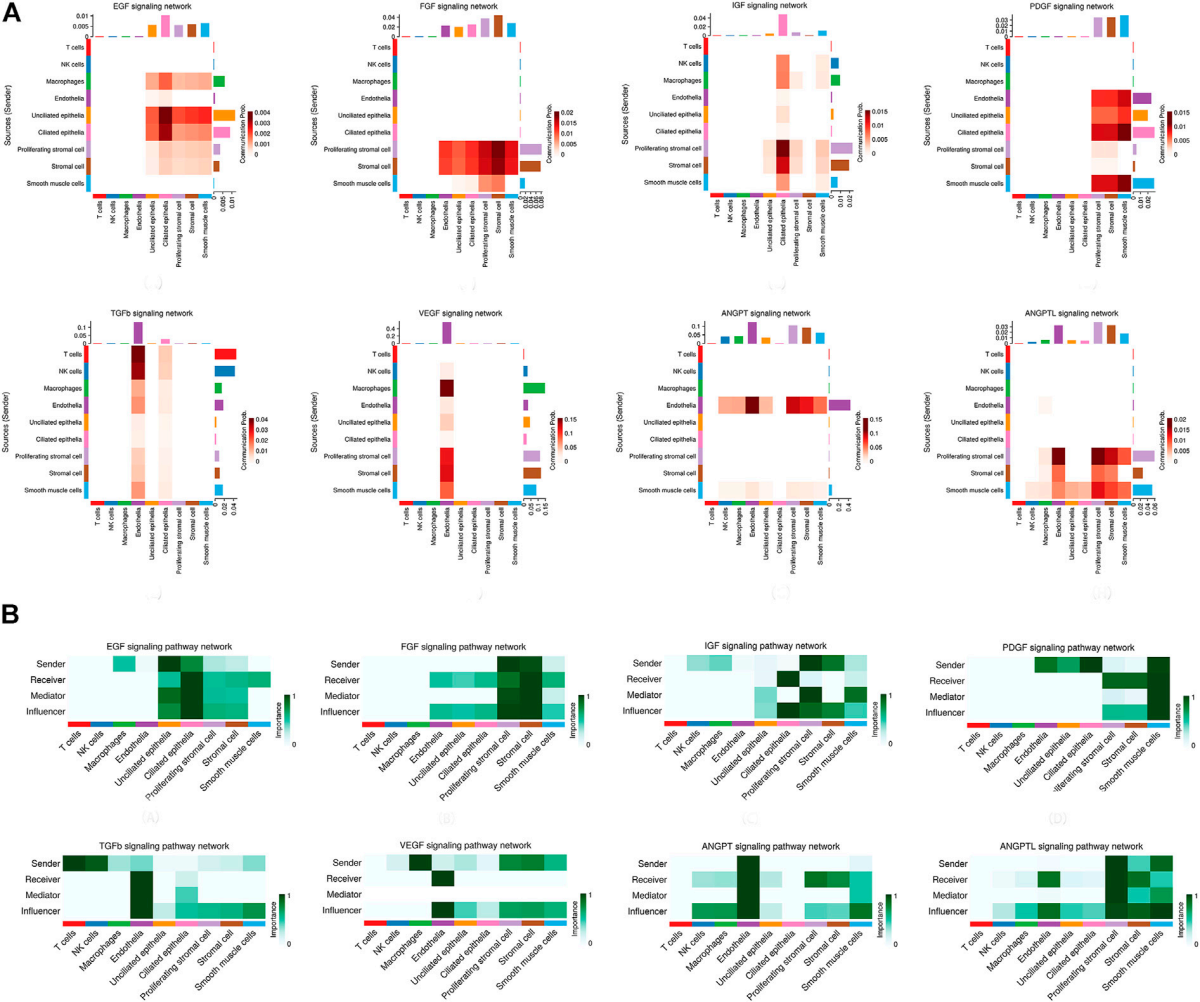
Heatmap of signaling pathways related to proliferation and angiogenesis and the relative importance of each cell group based on the computed network centrality measures of signaling networks. **(A)** For the heatmap of signaling pathways related to proliferation and angiogenesis. The communication probability of a signaling pathway was computed by summarizing the probabilities of its associated ligand–receptor pairs. The darker the color, the greater the communication probability between the two cell types. **(B)** For the relative importance of each cell group based on the computed network centrality measures of signaling networks. Influencer represents a kind of cell that can control information flow within a signaling network, and a higher value indicates greater control on the information flow. Gatekeeper represents a kind of cell that can control communication flow between any two cell groups, and a higher value indicates greater capability to control the communication flow. The meaning of importance is the magnitude of the possibility of four roles (sender, receiver, mediator, and influencer) that the cell types play. The darker the color, the greater the role cells play.

**FIGURE 4:**
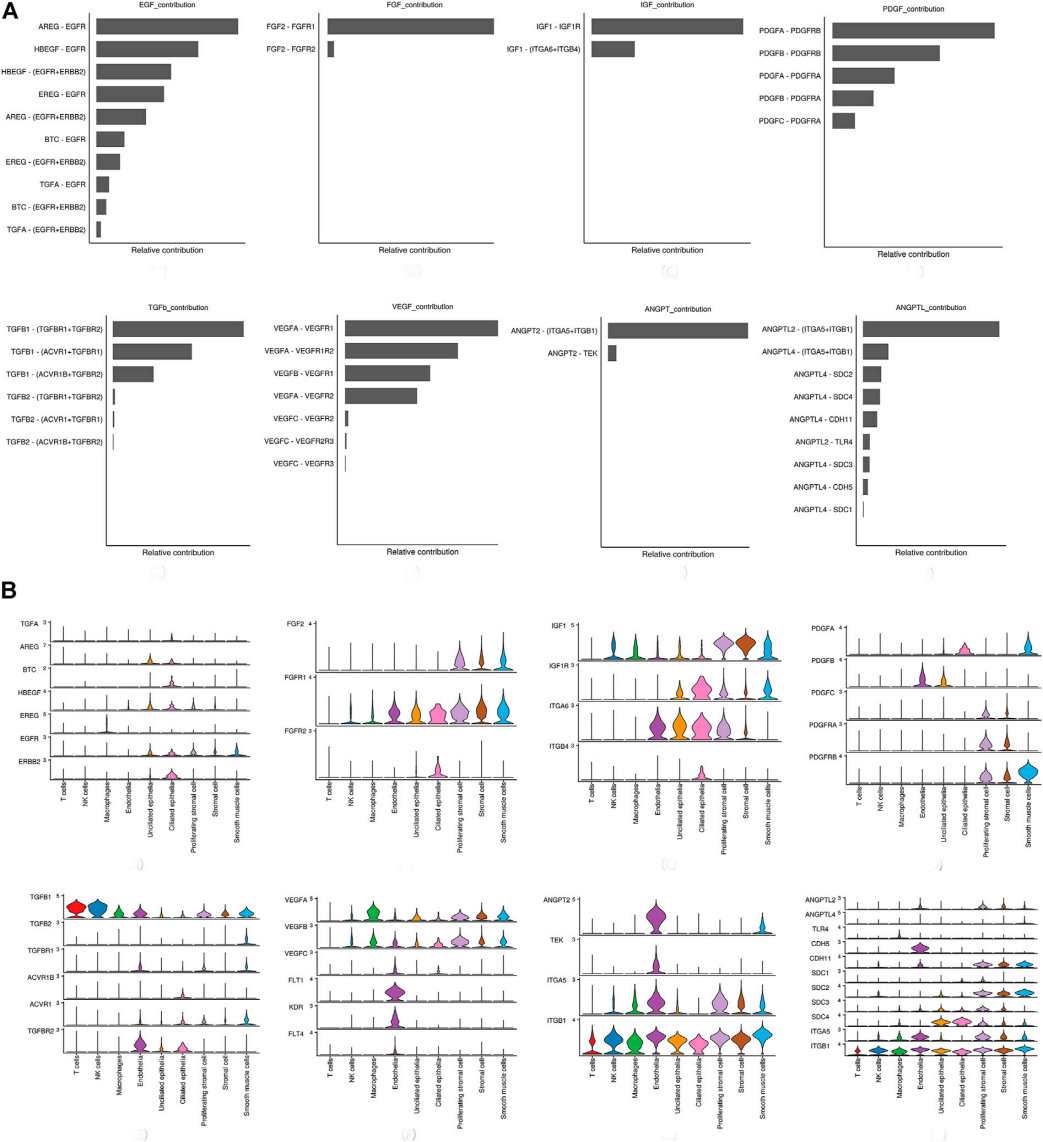
Relative contribution of each ligand–receptor pair to the overall communication network of signaling pathways and the expression patterns of signaling genes involved in the inferred signaling network. **(A)** Relative contribution of each ligand–receptor pair to the overall communication network of signaling pathways, which is the ratio of the total communication probability of the inferred network of each ligand–receptor pair to that of signaling pathways. **(B)** Violin plot showing the expression patterns of signaling genes involved in the inferred signaling network. Normalized expression levels are shown in the violin plot.

**FIGURE 5:**
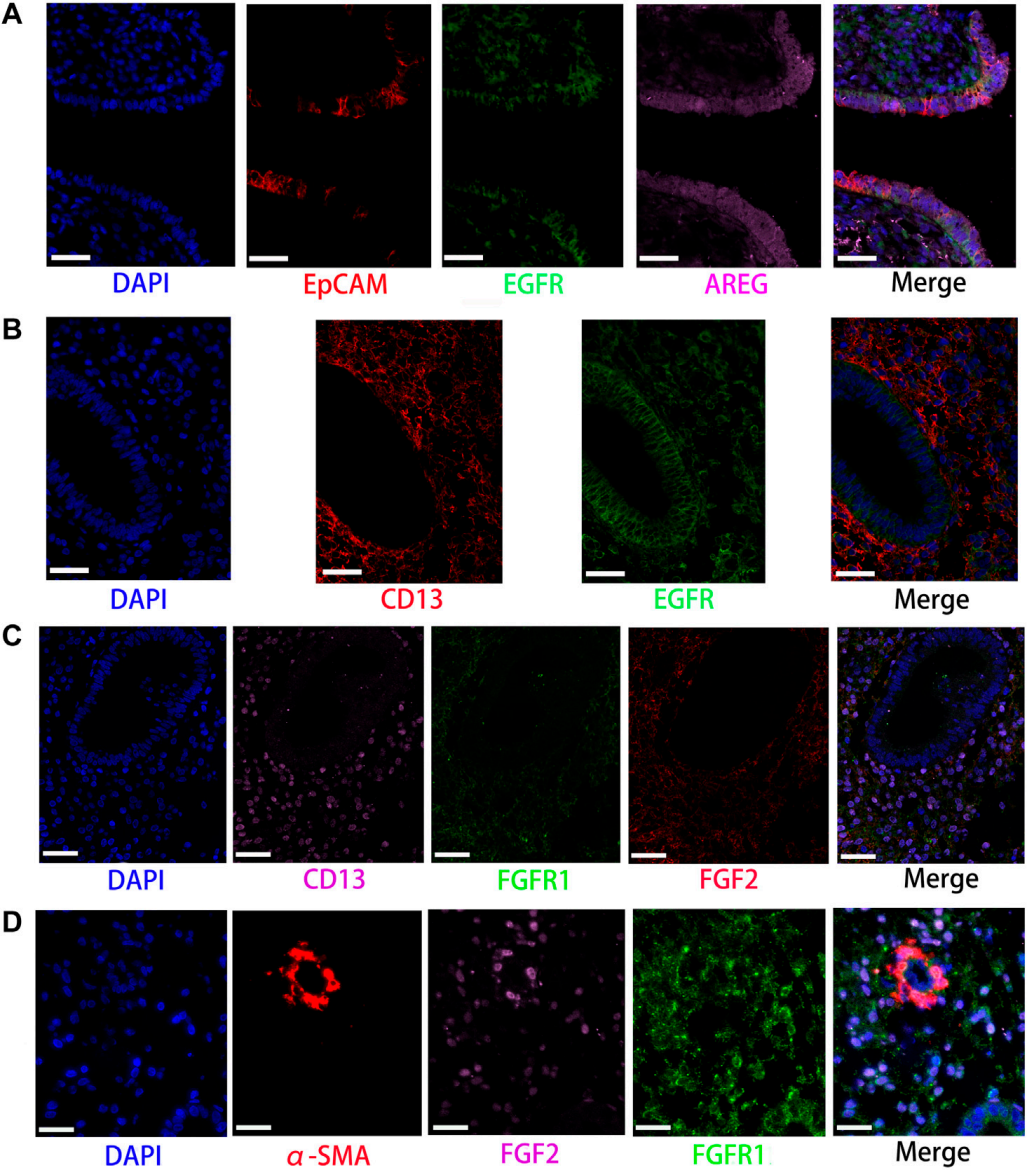
Partly ligand and receptor immunofluorescence staining of EGF and FGF signaling pathway. **(A)** Co-staining of EpCAM (epithelia, red) with EGF receptor (green), AREG (pink), and nucleus (blue) by immunofluorescence. **(B)** Co-staining of CD13 (stromal cells, red) with EGF (green) and nucleus (blue) by immunofluorescence. **(C)** Co-staining of CD13 (stromal cells, pink) with FGFR1 (green), FGF2 (red), and nucleus (blue) by immunofluorescence. **(D)** Co-staining of α-SMA (smooth muscle cells, red) with FGFR1 (green), FGF2 (pink), and nucleus (blue) by immunofluorescence. (Scale bars = 50 μm).

Network centrality analysis of the inferred EGF signaling network identified that stromal cells, proliferating stromal cells, ciliated epithelia, unciliated epithelia, and smooth muscle cells exhibited the properties of target cells (the receiver) in the EGF signaling pathway. On the other hand, the abovementioned five target cell types except for smooth muscle cells represented dominant source cells (the sender) of the EGF signaling pathway, which suggested these cells acted through the form of paracrine and autocrine signaling. In addition, macrophages also exhibited the properties of source cells in the EGF signaling pathway. Notably, T cells, NK cells were not found to interact with other cell types through the EGF signaling pathway. The results are shown in Figure 2B. The heatmap of the EGF signaling pathway showed a strong inter-association between ciliated epithelia and unciliated epithelial cells, as shown in Figure 3A. In addition, ciliated epithelia were the dominant mediator and influencer, suggesting their role as gatekeepers of cell–cell communication in the EGF signaling pathway, as shown in Figure 3B. In terms of ligand–receptor pairs, a total of 10 ligand–receptor pairs were found. Notably, three ligand–receptor pairs, namely, AREG-EGFR, HBEGF-EGFR, and HBEGF-(EGFR + ERBB2) were the dominant contributors to this communication network, as shown in Figure 4A. The corresponding violin plot showed the expression patterns of signaling genes involved in the EGF signaling network, and what is interesting is that ciliated epithelia specifically expressed ERBB2, as shown in Figure 4B. Compared to other cell types, the top 1 ligand–receptor pair AREG-EGFR in epithelia was noticeably expressed, which was validated using immunofluorescence staining (see Figure 5A). In addition, both stromal cells also expressed EGFR receptors, which was confirmed in our study (see Figure 5B).

### Fibroblast Growth Factor Signaling Pathway

For the FGF signaling pathway, both stromal cells became the main signal source cells and the same as smooth muscle cells. Moreover, both stromal cells, epithelia, smooth muscle cells, and endothelial cells are the main target cells. However, immune cells (T cells, NK cells, and macrophages) did not interact with other types of cells through the FGF signaling pathway. In addition, both stromal cells acted through the form of paracrine and autocrine signaling. The results are shown in Figure 2B. Compared to other cell types, there was a strong interconnection between stromal cells and proliferating stromal cells through the FGF signaling pathway, as shown in the heatmap (Figure 3A). Furthermore, both stromal cells were the prominent mediator, suggesting its role as gatekeepers of cell–cell communication, as shown in Figure 3B. Of note, the FGF2-FGFR1 ligand–receptor pair occupied an absolute dominance in the relative contribution of ligand–receptor pairs of the FGF signaling pathway, followed by the FGF2-FGFR2 ligand–receptor pair, as shown in Figure 4A. The corresponding violin plot showed the expression patterns of signaling genes involved in the FGF signaling network, and both stromal cells showcased the obvious FGF2-FGFR1 ligand–receptor pair, as shown in Figure 4B. Furthermore, we validated the FGF2-FGFR1 ligand–receptor pairs of both stromal cells and smooth muscle cells; the results are shown in Figure 5C.

### Insulin-like Growth Factor Signaling Pathway

**FIGURE 6:**
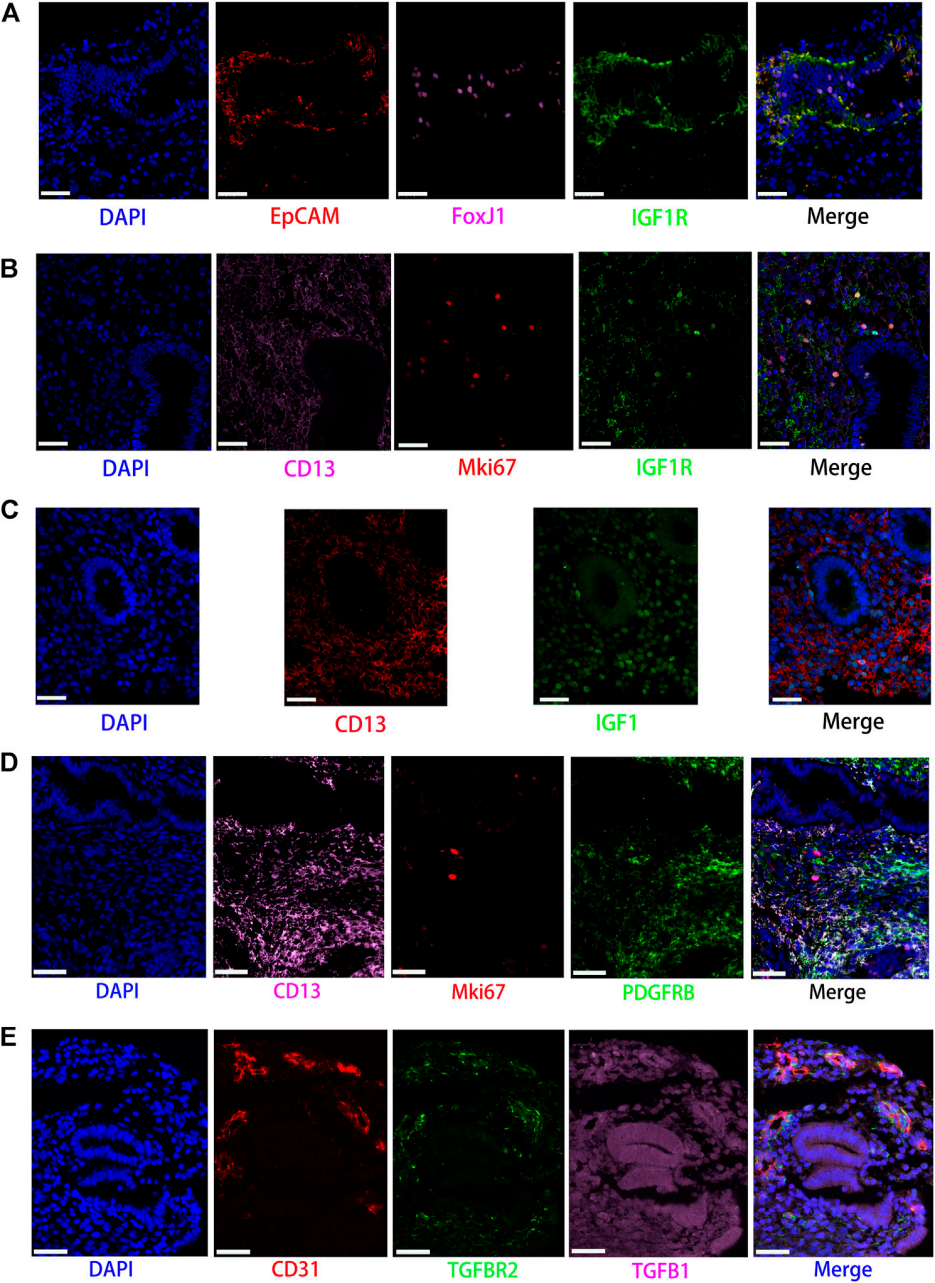
Partly ligand and receptor immunofluorescence staining of the IGF, PDGF, as well as TGFb signaling pathway. **(A)** Co-staining of EpCAM (epithelial cell, red) with FoxJ1 (Cilia, pink), IGF1R (green), and nucleus (blue) by immunofluorescence. **(B)** Co-staining of CD13 (stromal cell, pink) with Mki67 (red), IGF1R (green), and nucleus (blue) by immunofluorescence. **(C)** Co-staining of CD13 (stromal cell, red) with IGF1 (green) and nucleus (blue) by immunofluorescence. **(D)** Co-staining of CD13 (stromal cells, pink) with MKI67 (red), PDGFRB (green), and nucleus (blue) by immunofluorescence. **(E)** Co-staining of CD31 (endothelial cell, red) with TGFBR2 (green), TGFB1 (pink), as well as nucleus (blue) by immunofluorescence. (Scale bars = 50 μm).

Our data suggested that ciliated epithelia were the primary target cells for the IGF signaling pathway. Furthermore, both stromal cells, as the main sender of IGF signals, act significantly on the ciliated epithelia. Similarly, the IGF signaling pathway can act in the form of autocrine/paracrine. It is noteworthy that the IGF signaling pathway-related gene expression in the T cells was not detected, which insinuated that T cells may not function through the IGF signaling pathway. The results are shown in Figure 2B. The heatmap showed that there were strong interactions between proliferating stromal cells and ciliated epithelia, as shown in Figure 3A. In addition, proliferating stromal cells became the mediator of the IGF signaling pathway, which suggested that it was a gatekeeper of cell–cell communication, as shown in Figure 3B. In addition, only two ligand–receptor pairs IGF1-IGF1R and IGF1-(ITGA6 + ITGB4) were detected in the IGF signaling pathway. The relative contribution of the IGF1-IGF1R ligand–receptor pair was more than three times compared with IGF1-(ITGA6 + ITGB4), as shown in Figure 4A. The violin plot showed the expression patterns of signaling genes involved in the IGF signaling network, as shown in Figure 4B, and it was clear that the ciliated epithelia and proliferating stromal cells highly expressed IGF1R receptor and that both stromal cells highly expressed the IGF1 ligand, which were congruent with our validation experiment (see Figures 6A–C).

### Platelet-derived Growth Factor Signaling Pathway

CellChat analysis of the communication between endometrial cells revealed that the PDGF signaling pathway may be involved in the proliferation of the endometrium. Three kinds of cell types (stromal cells, proliferating stromal cells, and smooth muscle cells) were inferred to be the target cells of this signaling pathway. Except for immune cells (NK cells, T cells, and macrophages), all cell types played the role of signaling source cells. For both stromal cell populations, they acted through the form of bidirectional forward and reverse signals. The results are shown in Figure 2B. The heatmap of the PDGF signaling pathway showed smooth muscle cells, and ciliated epithelia have a strong interaction with themselves, as shown in Figure 3A. In addition, smooth muscle cells were the prominent mediator and influencer controlling the communications, as shown in Figure 3B. As for ligand–receptor pairs, there are five ligand–receptor pairs included in the PDGF signaling pathway. In order of the relative contribution degree from high to low, these ligand–receptor pairs were successively PDGFA-PDGFRB, PDGFB-PDGFRB, PDGFA-PDGFRA, PDGFB-PDGFRA, and PDGFC-PDGFRA, as shown in Figure 4A, and their violin plot showing the expression patterns of signaling genes is shown in Figure 4B. It is worth noting that the inferred results indicated proliferating stromal cells also expressed PDGFRB, which was furtherly validated in the immunofluorescence experiment (see Figure 6D).

### Transforming Growth Factor b Signaling Pathway

Network centrality analysis of the inferred TGFb signaling network identified that all cell populations are sources of TGFb ligands. Importantly, CellChat also predicted that endothelial cells and ciliated epithelia significantly contributed to TGFb signal production in the proliferation of the endometrium, which revealed that the TGFb signaling network in proliferation is complex and highly redundant with multiple ligand sources targeting a large portion of endothelial cells and ciliated epithelia. Interestingly, CellChat showed that the majority of TGFb interactions among cells are paracrine, with only endothelial cells and ciliated epithelia demonstrating prominent autocrine signaling. The results are shown in Figure 2B. The heatmap of the TGFb signaling pathway showed that there were strong associations between endothelial cells and two immune cells (NK cells and T cells), as shown in Figure 3A. Furthermore, endothelial cells were also the dominant mediator and influencer, suggesting their role as a gatekeeper of cell–cell communication, as shown in Figure 3B. Notably, TGFb signaling was dominated by the TGFB1 ligand and its multimeric TGFBR1/TGFBR2 receptor among all known ligand–receptor pairs, as shown in Figure 4A. The violin plot showed a significant expression of TGFB1 gene in all cell types, as shown in Figure 4B, and salient expression of TGFBR2 was found in endothelial cells, which were furtherly validated by our immunofluorescence result (see Figure 6E).

### Vascular Endothelial Growth Factor Signaling Pathway

**FIGURE 7:**
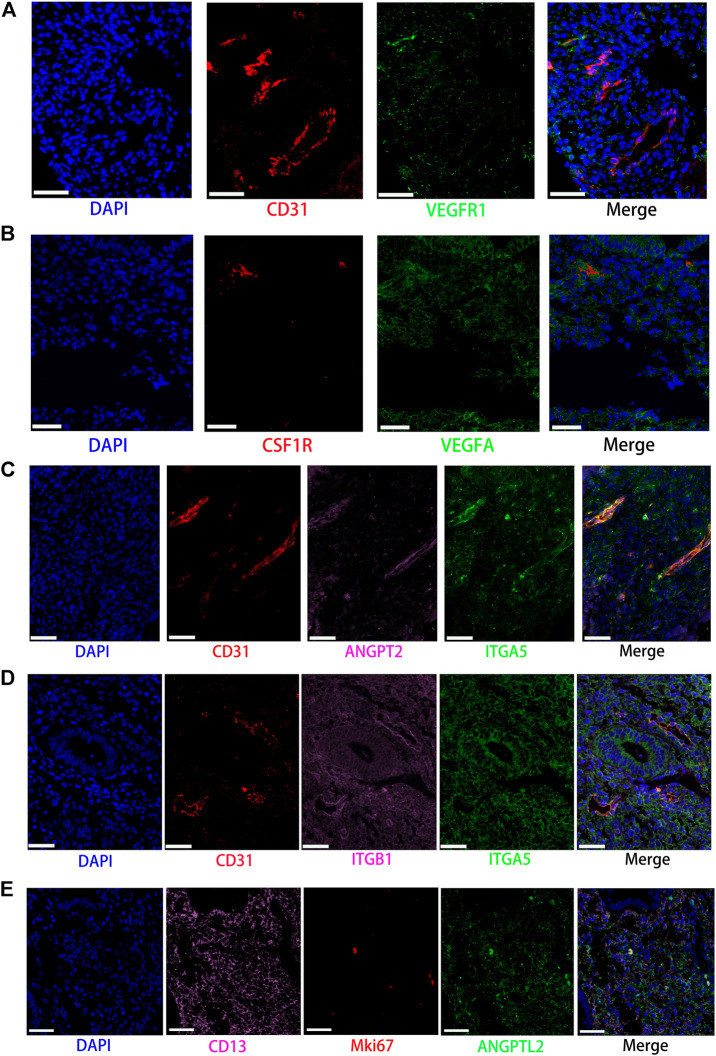
Partly ligand and receptor immunofluorescence staining of VEGF, ANGPT, as well as ANGPTL signaling pathway. **(A)** Co-staining of CD31 (endothelial cell, red) with VEGFR1 (green) and nucleus (blue) by immunofluorescence. **(B)** Co-staining of CSF1R (macrophage, red) with VEGFA (green) and nucleus (blue) by immunofluorescence. **(C)** Co-staining of CD31 (endothelial cell, red) with ANGPT2 (pink) and ITGA5 (green) as well as nucleus (blue) by immunofluorescence. **(D)** Co-staining of CD31 (endothelial cell, red) with ITGB1 (pink) and ITGA5 (green) as well as nucleus (blue) by immunofluorescence. **(E)** Co-staining of CD13 (stromal cell, pink) with MKI67 (red) and ANGPTL2 (green) as well as nucleus (blue) by immunofluorescence. (Scale bars = 50 μm).

In contrast with other signaling pathways, CellChat analysis of the inferred VEGF signaling network revealed its very distinct, nonredundant structure with only one target cell population endothelial cells regulated largely by most endometrial cells. Moreover, endothelial cells also acted in the autocrine form. Nevertheless, T cells did not establish connections with endothelial cells through the VEGF signaling pathway. The results are shown in Figure 2B. Compared with other cell types, the association of macrophages with endothelial cells through the VEGF signaling pathway was more significant, followed by stromal cells, as shown in Figure 3A. It is unexpected that network centrality analysis confirmed that endothelial cells were the prominent influencer controlling the communications, as shown in Figure 3B. Among all known ligand–receptor pairs, VEGF signaling was dominated by ligand VEGFA and its receptor VEGFR1, and the second was ligand VEGFA and its receptor VEGFR1R2. The relative contribution of ligand VEGFB was less than that of ligand VEGFA but was significantly higher than that of ligand VEGFC. The results are shown in Figure 4A. Violin plots showing the expression patterns of signaling genes involved in the VEGF signaling network are shown in Figure 4B, and noticeable VEGFR1 (FLT1) expression in endothelial cells and VEGFA expression in macrophages was also demonstrated in our study (see Figures 7A,B).

### Angiopoietin Signaling Pathway

Network centrality analysis of the inferred ANGPT signaling network identified that endothelial cells and smooth muscle cells were only two sources for ANGPT ligands acting on almost all cell types, except for T cells and ciliated epithelia. Intriguingly, the two cell populations (endothelial cells and smooth muscle cells) also exhibited autocrine signaling, as shown in Figure 2B. The heatmap of the ANGPT signaling pathway showed endothelial cells have strong associations with themselves, as shown in Figure 3A. Furthermore, endothelial cells were also the dominant mediator and influencer of the ANGPT signaling pathway, suggesting its role as a gatekeeper of cell–cell communication (see Figure 3B). Significantly, merely the two ligand–receptor pairs were found among all known ligand–receptor pairs, that is, ANGPT signaling was dominated by the ANGPT2 ligand and its multimeric ITGA5/ITGB1 receptor, followed by the ANGPT2-TEK ligand–receptor pair, as shown in Figure 4A. The violin plot shows the expression patterns of signaling genes involved in the ANGPT signaling network, as shown in Figure 4B, and it can be clearly seen that endothelial cells highly expressed ANGPT2 and receptor complex ITAG5 + ITGB1, which were corroborated in the present work (see Figures 7C,D).

### Angiopoietin-Like Protein Signaling Pathway

For the ANGPTL signaling pathway, smooth muscle cells, as the primary sender of ANGPTL signals, were associated with almost all cell types of the endometrium. Except for smooth muscle cells, both stromal cells also acted as signaling senders and were associated with multiple cells in the endometrium. But, endothelial cells were the main receiver of the ANGPTL signaling pathway. The interaction strength between endothelial cells and proliferating stromal cells *via* the ANGPTL signaling pathway is higher than that between other cell types. Similar to the ANGPT signaling pathway, T cells also did not establish connections with other cell types through the ANGPTL signaling pathway. The results are shown in Figure 2B. Compared with other cell types, the intense association between the proliferating stromal cells and the endothelial cells and between the proliferating stromal cells with themselves was exhibited, as shown in Figure 3A. It is to be noted that proliferating stromal cells were the potential dominant mediator, suggesting its possible role as a gatekeeper of cell–cell communication, as shown in Figure 3B. Notably, two ligand–receptor pairs, namely, ANGPTL2-ITGA5/ITGB1 and ANGPTL4-ITGA5/ITGB1 were the dominant contributors to this communication network, as shown in Figure 4A. The violin plot showing the expression patterns of signaling genes involved in the ANGPTL signaling network is shown in Figure 4B. Compared with other cell types, proliferating stromal cells highly expressed ANGPTL2, which was substantiated in our study (see Figure 7E).

### Communication Patterns

Cross-referencing outgoing and incoming signaling patterns provided a quick insight into the autocrine-acting vs. paracrine-acting pathways of a given cell type. CellChat analysis on these cells identified that different cells might act as a dominant communication “hub” in different signaling pathways, which sent and received signals *via* different quantities of ligand–receptor pairs. In the present study, stromal cells and proliferating stromal cells represented cell–cell interaction hubs with a large number of EGF, PDGF incoming signals, and FGF outgoing signals. Yet, endothelial cells exhibited cell–cell interaction hubs with plenty of VEGF, TGFb incoming signals, and ANGPT outgoing signals. Furthermore, unciliated epithelial cells, ciliated epithelial cells, and macrophages exhibited cell–cell interaction hubs with substantial EGF outgoing signals. In addition, ciliated epithelial cells represented cell–cell interaction hubs with a large number of IGF and TGFb incoming signals. Also, smooth muscle cells represented lots of PDGF incoming signals and ANGPT and ANGPTL outgoing signals.

**FIGURE 8:**
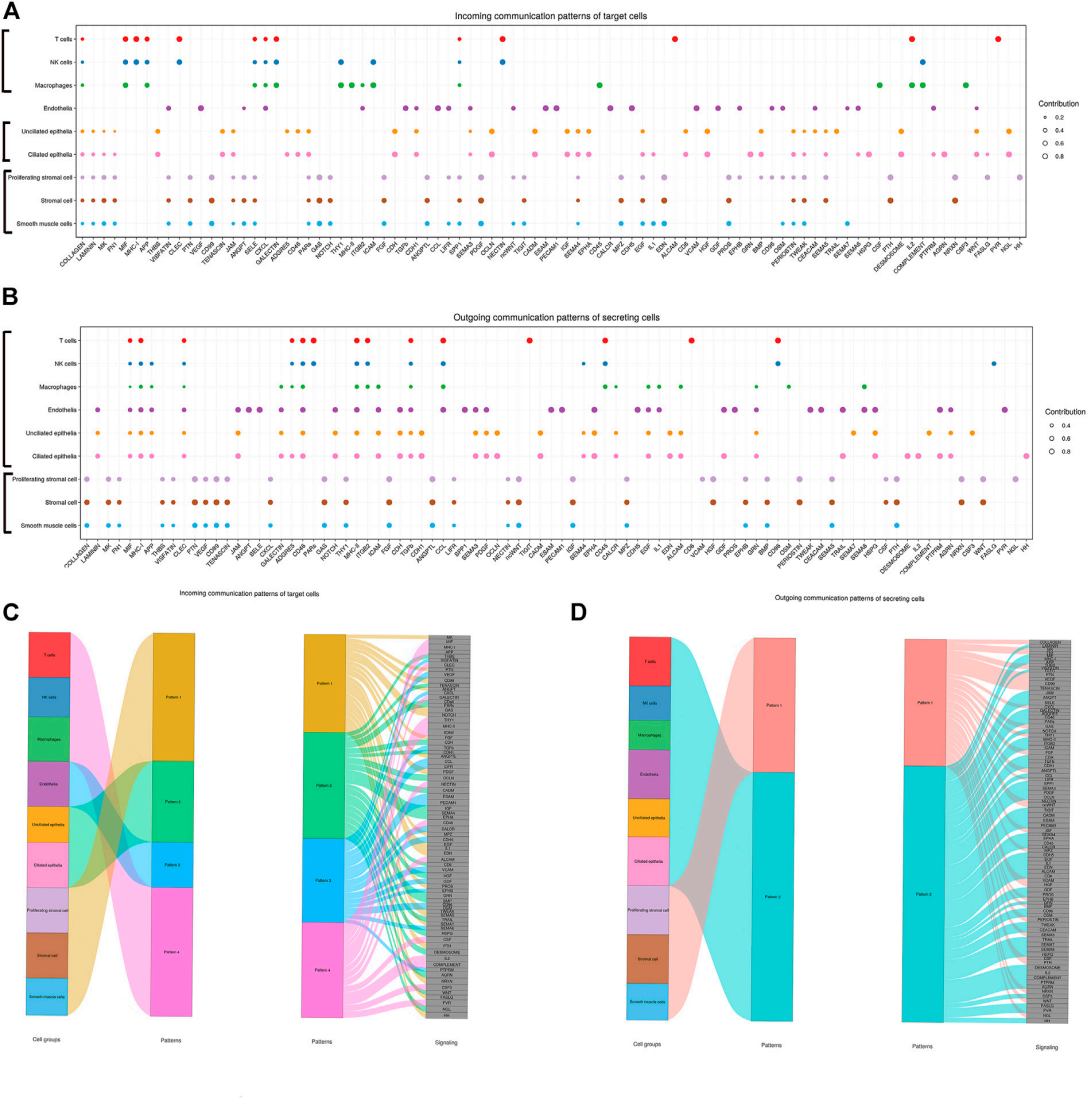
Inferred incoming and outgoing communication patterns of endometrial cells. Incoming patterns show how the target cells (i.e., cells as signal receivers) coordinate with each other as well as how they coordinate with certain signaling pathways to respond to incoming signals. Outgoing patterns reveal how the sender cells (i.e., cells as signal sources) coordinate with each other as well as how they coordinate with certain signaling pathways to drive communication. Simply put, cell communication patterns are classified according to the similarity of cell communication. **(A)** Dot plot exhibited incoming communication patterns of target cells. **(B)** Dot plot exhibited outgoing communication patterns of secreting cells. **(C)** Inferred incoming communication patterns of target cells. Incoming patterns show how the target cells coordinate with each other as well as how they coordinate with certain signaling pathways to respond to signals. **(D)** Inferred outgoing communication patterns of secreting cells, which show the correspondence between the inferred latent patterns and cell groups, as well as signaling pathways. The thickness of the flow indicates the contribution of the cell group or signaling pathway to each latent pattern.

In addition, an important question is how multiple cell groups and signaling pathways coordinate to function. To address this question, CellChat used a pattern recognition method based on non-negative matrix factorization to identify the global communication patterns as well as the key signals in different cell groups. In our study, the application of this analysis uncovered four patterns for incoming signaling and two patterns for outgoing signaling. The communication patterns of target cells showed that incoming signaling of both stromal cells and smooth muscle cells was driven by pattern #1, which includes signaling pathways such as FGF and ANGPT. Both epithelia were dominated by pattern #2, which represented multiple pathways, including but not limited to IGF and WNT. Endothelial cells were characterized by Pattern #3, driven by VEGF and TGFb pathways, etc. In addition, all incoming signaling of immune cells was characterized by Pattern #4, representing pathways such as IL2 and MIF. On the other hand, this output revealed that outgoing signaling of both stromal cells and smooth muscle cells were characterized by pattern #1, which represented multiple pathways, including but not limited to VEGF and ANGPTL. Yet, both epithelial and endothelial cells and immune cells were dominated by pattern #2 representing multiple pathways, including but not limited to ANGPT and TGFb. The results are shown in Figure 8.

## Discussion

Analysis of the process of regeneration and angiogenesis in the human endometrium would provide useful information for the fields of reproductive biology, regenerative medicine, and tissue engineering. During each menstrual cycle, a new vascular system is developed to support cellular growth and differentiation (Jabbour et al., 2006). Moreover, growth factors and cytokines are paramount to endometrial proliferation (Wang et al., 2020). In addition, the development of the vascular system in the human endometrium is believed to be orchestrated by the coordinated interactions of several angiogenic factors (Girling and Rogers, 2005), including vascular endothelial cell growth factor (VEGF), soluble VEGF receptor 1 (sVEGFR-1), and angiopoietin (ANGPT) (Koga et al., 2001; Albrecht and Pepe, 2003). However, the endometrium, a complex multiple-cellular component tissue, secreted a wide variety of cytokines and growth factors, which were regulated by estrogen in the proliferative phase and formed a complex network. So far, the network of endometrial regeneration has still not been fully defined.

The CellChat R package is a versatile and easy-to-use toolkit for inferring, analyzing, and visualizing cell–cell communication from any given scRNA-seq data (Jin et al., 2021). In the present study, we reported the signaling ligand–receptor interactions that consider the multimeric structure of ligand–receptor complexes and additional effects on the core interaction by soluble and membrane-bound stimulatory and inhibitory cofactors using CellChat. We revealed the intercellular link by analyzing the proliferative phase endometrium. Based on this model, the cell–cell communication network of endometrial cells was inferred, and some interesting aspects will be discussed below.

In the following text, we first presented the main expression patterns starting with a comparison of tissue-level transcriptomes to the sum total of single-cell transcriptomes and then characterized the clusters by their signatures relative to the particular assembly of cells. Compared with the previous study (Rutanen, 1998), more cell types, particularly proliferating stromal cells and ciliated epithelial cells, were annotated in our study. The second part of the study focused on the cell–cell communication network and contributions to pathways known to play major roles at the uterine surface by computer predictions and mathematical and network analysis. The third part focused on a large number of receptor–ligand pairs, which were excavated and further divided into 88 signaling pathways. Moreover, the signaling pathways highly associated with endometrial regeneration were selected for further analysis and validation.

### For the Epidermal Growth Factor Signaling Pathway

Studies indicated that much of the mitotic activity is mediated *via* the expression of EGF and its receptor. Furthermore, EGF and its receptor (EGFR) have been found in human endometrium and human EGF Receptor 1 showed the highest expression during the proliferative phase (Ushiro et al., 1996). In addition, EGF is ascribed with a variety of biological effects including mitogenic effect, stimulating proliferation of many cell types including human microvascular endothelial cells and lymphatic endothelial cells (Möller et al., 2001). Our study found unciliated epithelial cells and ciliated epithelial cells performed as the main sender of the EGF signaling pathway and the same with stromal cells, which were in agreement with the previous studies (Möller et al., 2001; Aghajanova et al., 2008). However, it is of particular note that ERBB2 was specifically expressed in ciliated epithelial cells, which was not reported before. Recent data indicated that AREG, a member of the EGF family, can be expressed by a variety of activated immune cells (including T cells) under various inflammatory conditions, mainly by promoting the restoration of tissue integrity after acute or chronic inflammation-related injury (Zaiss et al., 2015). In addition, the study investigating decidual tissue of early-pregnancy phase by single-cell sequencing has also revealed that NK cells can be interconnected with stromal cells *via* AREG-EGFR ligand–receptor pairs (Vento-Tormo et al., 2018). However, our study did not detect that T cells and NK cells interacted with other cell types through the EGF signaling pathway, but macrophages. The plausible explanation is the presence of tissue specificity or the variations of cell communication with the cycle phases or the very low expression of AREG in T cells and NK cells.

### For the Fibroblast Growth Factor Signaling Pathway

FGF family have emerged as multifunctional regulators of cellular processes implicating differentiation, migration, and cell growth (Powers et al., 2000). One of its most famous members, FGF-2 (also called bFGF), expressed in a wide variety of adult and fetal tissues, is involved in the proliferation of fibroblasts and endothelial cells, migration, and differentiation processes (Smith, 1995). Sangha et al. (1997) identified the strongest hybridization for FGF-2 mRNA in proliferative stromal cells. FGF-2 also became evident in the luminal epithelium and the stromal matrix. FGF-2 mRNA was highly expressed during the late proliferative stage. At the moment, one may speculate that the binding of FGF-2 leads to the internalization of the FGF-2/FGFR1 complex which is involved in the control of cell growth and proliferation of endometrial stromal and epithelial cells by regulating gene activity in response to steroids (Welter et al., 2004). FGFR1 has been detected in distinct isoforms in the human endometrium. Consistent with previous studies, FGF-2/FGFR1 is also the primary receptor–ligand pair in our study. However, our study inferred more cell–cell communication relationships of the FGF signaling pathway than those in previous studies. Especially, stromal cells and proliferating stromal cells presented conspicuous communication relationships of the FGF signaling pathway by autocrine signaling, which signified the FGF signaling pathway may have played a prominent role in the proliferation of endometrial stromal cells. In addition, similar to previous studies (Cordon-Cardo et al., 1990; Giudice, 1994; Edwards et al., 2011; Chiumia et al., 2020), we also found that FGF2 and FGFR1 were localized in smooth muscle cells, suggesting that FGF may participate in angiogenesis in the proliferative endometrium. In addition, our study found that FGFR2 was specifically expressed in the ciliated epithelial cells but not the unciliated epithelial cells of proliferative endometrium, which was not reported before. By contrast, immune cells may not function through the FGF signaling pathway in endometrium.

### For the Insulin-like Growth Factor Signaling Pathway

IGFs are believed to play an important role in endometrial proliferation and differentiation and in embryo–endometrial interaction (Rutanen et al., 1988). Studies indicated the mRNA encoding IGF-I is most abundant in the late proliferative phase, supporting the hypothesis that endometrial IGF-I mRNA expression is estrogen-dependent and that IGF-I mediates the estrogen effect (Murphy and Ghahary, 1990; Giudice et al., 1993). In addition, the IGF system has autocrine and paracrine functions in the regulation of endometrial proliferation (Rutanen, 1998), which was also confirmed by our results. In addition, compared with previous studies, our data manifested that IGF signaling may function through both ligand–receptor pairs, IGF1-IGF1R, and IGF1-(ITGA6 + ITGB4). Notably, ciliated epithelial cells were the target cells of IGF signaling pathways of multiple cell types and ciliated epithelial cells specifically expressed the ITGB4 receptor, suggesting that ciliated epithelial cells may appreciably function through the IGF signaling pathway.

### For the Platelet-derived Growth Factor Signaling Pathway

PDGF-mediated signaling plays an essential role in cellular proliferation, migration, angiogenesis, and tissue injury and in its repair (Ariyanti et al., 2017). Distinct PDGF isoforms have been shown to stimulate proliferation and migration of endometrial stromal cells *in vitro*, indicating that PDGF could help promote endometrial tissue repair (Matsumoto et al., 2005; Annunziata et al., 2012). It was recently reported that platelet-derived soluble factors could promote migration and proliferation of endometrial epithelial cells *in vitro* (Matsumoto et al., 2005). To some extent, our results were also in favor of the abovementioned results. Our study found that stromal cells, proliferating stromal cells, and smooth muscle cells were the target cells of the PDGF signaling pathway, especially stromal cells and proliferating stromal cells were appreciably regulated by other cell types, which suggested that both stromal cells might be the primary cell types that control endometrial proliferation through the PDGF signaling pathway. In addition, of particular note is PDGFRB, a putative endometrial stem cell gene, which was pronouncedly expressed in proliferating stromal cells. In addition, the stemness-related genes CD146, CD105, and CD90 were also expressed in proliferating stromal cells (the results were not shown), suggesting that the kind of cell may have a potential for stemness, which requires further validation.

### For the Transforming Growth Factor b Signaling Pathway

Studies found TGFb is implicated in gene expression, cell motility, proliferation, apoptosis, differentiation, immune responses, and tumorigenesis (Seoane and Gomis, 2017). Transforming growth factor-β1 (TGF-β1) plays a crucial role in inducing and promoting the differentiation and proliferation of mesenchymal cells, in the secretion of extracellular matrix-associated components, and is a major cytokine in initiating and terminating tissue repair downstream of the TGF-β/Smad signaling pathway. Furthermore, the role of TGF-β1 has been demonstrated to be potentially regulated by a variety of cytokines, hormones, enzymes, and microRNAs (Abudukeyoumu et al., 2020). TGF-β1 involves in the body’s inflammatory response and tissue repair and regulates cell growth and differentiation (Distler et al., 2003). In our study, all cell populations were sources of TGFb ligands, which suggested that the TGFb signaling pathway is prevalent in the endometrium. Although the expression of TGFb receptors has been reported in epithelial cells, endothelial cells, stromal cells, and smooth muscle cells in previous studies (Jones et al., 2006; Lash et al., 2012), in contrast, we found that only two cell types, namely, ciliated epithelial and endothelial cells were the target cells of the TGFb signaling pathway. This may be due to our study targeting the whole ligand–receptor complex.

### For the Vascular Endothelial Growth Factor Signaling Pathway

Many studies have demonstrated VEGF expression in human endometrium. VEGF can stimulate endothelial cell proliferation, permeability, migration, and assembly into capillary tubes. VEGF is essential for the rapid burst of angiogenesis that occurs during postmenstrual repair and in the early proliferative phases in the primate endometrium and further plays a role in the re-epithelialization of the endometrium. Recent studies have reported that the role of VEGF in the early angiogenic processes is associated with postmenstrual regeneration of the endometrium (Fan et al., 2008). Furthermore, the vessel length density increased in consequence of the major increase in the average of the vessel segment length throughout the proliferative phase. The VEGF actions on angiogenesis processes were mediated through binding to the fms-like tyrosine kinase (VEGFR-1), the tyrosine kinase receptor (VEGFR-2), and neuropilin-1, which are generally found on vascular endothelial cells. However, the role of VEGFR-2 receptors in angiogenesis appeared to be more important than that of the other two receptors (Ferrara et al., 2003; Gellersen and Brosens, 2014). Different from the previous findings (Meduri et al., 2000; Sugino et al., 2002), our study found VEGF signaling was dominated by the VEGFA ligand and its VEGFR1 receptor in all known ligand–receptor pairs, and the second was VEGFA ligand and its VEGFR1R2 receptor. Endothelial cells were the only cells population expressing the VEGF receptor, which suggested that endothelial cells are likely involved in controlling the angiogenesis by the VEGF signaling pathway. Notably, macrophages had more significant regulatory effects on endothelial cells by the VEGF signaling pathway, compared to other cell types in our study. Other studies also found that there were associations between macrophages and endothelial cells (Vento-Tormo et al., 2018; Hernandez and Iruela-Arispe, 2020). Moreover, it was recently reported that VEGF receptor 1-expressing macrophages recruited from bone marrow enhanced angiogenesis in endometrial tissues (Sekiguchi et al., 2019). However, the study investigating decidual tissue has revealed that macrophages can associate with stromal cells *via* KDR-VEGFA ligand–receptor pairs (Vento-Tormo et al., 2018), which were not detected in the endometrium during the proliferative phase of our study.

### For the Angiopoietin Signaling Pathway

Angiopoietins comprised a key group in the promotion of angiogenesis and vessel remodeling in the endometrium. The balance between the expression of ANGPT1 and ANGPT2 is important for angiogenesis. ANGPT1 increases the association of endothelial cells with pericytes and vascular smooth muscle cells to stimulate the maturation of newly formed blood vessels. Conversely, ANGPT2 is a natural antagonist of ANGPT1 that initiates neovascularization (Nishigaki et al., 2011). Increases in the ANGPT2/ANGPT1 ratio are associated with new blood vessel formation. With regard to the effects of steroid hormones, E2 suppressed ANGPT1 production, resulting in an increase in the ANGPT2/ANGPT1 ratio in human endometrial stromal cells (Tsuzuki et al., 2013). ANGPT2 mediated β1-integrin (ITGB1) activation and elongated matrix adhesions and actin stress fibers, regulating vascular endothelial cells’ cadherin-containing cell–cell junctions (Hakanpaa et al., 2015). Indeed, our study suggested that ANGPT2 might act through the ANGPT2- ITGA5/ITGB1 ligand–receptor pair. On the other hand, ANGPT1-induced TIE2 (TEK) activation resulted in the enhancement of endothelial cell survival signals and the maintenance of endothelial cell barrier and quiescent vasculature. Conversely, ANGPT2 normally functions as an ANGPT1 antagonist and increases vascular permeability, destabilizes quiescent vasculature, and primes the endothelial cell bed for angiogenesis (Huang et al., 2010). Interestingly, ANGPT1 was not detected. Nevertheless, the ANGPT2 ligand and its multimeric receptor ITGA5/ITGB1 were markedly found in our study. In addition, endometrial ANGPT2-TEK ligand–receptor pair specifically presented in endothelial cells in our study, and the potential function of the ligand–receptor pair needs further investigation.

### For the Angiopoietin-Like Protein Signaling Pathway

Angiopoietin-like proteins (ANGPTLs) are a family of proteins structurally similar to angiopoietins. There were two ligands ANGPTL2 and ANGPTL4 found in our study. Kim et al. (1999) found that ANGPTL2 mRNA levels were the highest in blood vessels and skeletal muscle in rat embryos but highest in the heart, small intestine, spleen, and stomach tissue in adult humans, suggesting that a special role of ANGPTL2 might exist in vasculature development. In addition, they found that the exogenous addition of recombinant human ANGPTL2 induces sprouting of porcine pulmonary arterial endothelial cells (PPAECs) *in vitro* (Kim et al., 1999). ANGPTL4 is a secreted protein involved in the regulation of vascular permeability, angiogenesis, and inflammatory responses in different kinds of tissues. Inflammatory response associated with ANGPTL4 also leads to minimal change in glomerulonephritis and wound healing (Guo et al., 2014). Many studies have illustrated that ANGPTL4 acted as a multifunctional secretory protein and was involved in the regulation of lipid metabolism, wound healing, and angiogenesis in various tissues including the endometrium (Hato et al., 2008; Goh et al., 2010). However, as far as our knowledge is concerned, previous studies have not elucidated which type of cell acts through the ANGPTL signaling pathway in the endometrium. Our study suggested that smooth muscle cells might broadly regulate other cell types in the human endometrium through the ANGPTL signaling pathway. In addition, both stromal cells could also function by the ANGPTL signaling pathway. Similar to VEGF signaling, endothelial cells were also the main target cells, being regulated by three cell types (smooth muscle cells, stromal cells, and proliferating stromal cells). ANGPTL4 reduction has been shown to impair endometrial angiogenesis and endometrial receptivity in patients with recurrent implantation failure (Li et al., 2020), ANGPTL2 expression in the proliferative endometrium appeared to be reported for the first time in our study. Immunofluorescence analysis showed that ANGPTL2 was widely expressed in stromal cells, and the violin plot showed that the expression of ANGPTL2 was higher than that of ANGPTL4 in the proliferative endometrium, suggesting that its role in endometrial angiogenesis might be underestimated and deserved further study. In addition, the ligands ANGPT2 and ANGPTL2 shared the same ITGA5/ITGB1 receptor. However, a further investigation of their downstream signaling pathway was required.

### For Communication Pattern

CellChat communication pattern analysis can uncover coordinated responses among different cell types. Different cell types may simultaneously activate the same cell type–independent signaling patterns or different cell type–specific signaling patterns. Different numbers of patterns provide different resolutions when recovering coordinated responses. This analysis could potentially help derive general cell–cell communication principles. For incoming communication patterns, both stromal cells and smooth muscle cells shared the same communication pattern (Pattern 1). Both epithelia shared another communication pattern (Pattern 2). Similarly, immune cells (T cells, NK cells, and macrophages) also shared the same communication pattern (Pattern 4). Different from other cell types, endothelial cells exclusively used a communication pattern (Pattern 3). For outgoing communication patterns, only two patterns were found. Both stromal cells and smooth muscle cells shared the same communication pattern (Pattern 1) and the rest cell types shared another communication pattern (Pattern 2), which suggested that outgoing communication patterns were less different than incoming communication patterns. Taken together, CellChat analysis on joint scRNA-seq datasets enables the multifaceted assessment of intercellular communication patterns.

### Advantages and Limitation

Cell interactions are part of the core biological function of endometrial tissues (Hernandez and Iruela-Arispe, 2020). The accuracy of the assigned roles for the signaling molecules and their interactions is crucial for predicting biologically meaningful intercellular communications. Though the inferred cell communication network remains hypothetical, the coexpression of ligands and receptors in adjacent cells is more convincible for functional importance than the gene expression of a single-cell type. CellChat’s predictions can recapitulate known biology to a substantial degree; therefore, CellChat performs well at predicting stronger interactions, which is helpful for narrowing down on interactions for further experimental validations. Furthermore, it will be interesting to broaden this approach in the future with additional cell types, in particular myometrial cells, and earlier gestational stages, as well as to use these results as a standard against which to interpret disease-related deviations.

Nevertheless, systematic evaluation of predicted cell–cell communication networks is challenging due to the lack of ground truth. Of note, failed detection of interactions with multi-subunits might be also caused by low expression of multi-subunits of the receptors that are not captured using scRNA-seq. Although we tried our best to validate the signaling pathways of interest, when discussing our cell–cell network model, it is important to note that this model was inferred from RNA expression rather than the presence of corresponding proteins, which was dependent upon the degree of posttranscriptional regulation. In addition, except the eight signaling pathways selected in this study, there were still many signaling pathways pertinent to endometrial growth and angiogenesis that were not further analyzed and validated in our study. In addition, although few data are available, it would be interesting and meaningful to compare the differences of communication networks among different phases of the menstrual cycle endometrium as well as early pregnancy decidua, which requires further study. Notwithstanding, our results still broadened the dimension of study in endometrial proliferation and angiogenesis.

## Summary

Our study provided a rich compendium resource of gene expression profiles of cell–cell interactome among endometrial cells and discovered some novel intercellular communications as well as built cell–cell communication atlases. The results indicated that cell–cell interactome involved fine-tuned interactions *via* numerous pathways. Different cell types can function in the proliferation of the endometrium through a variety of signaling pathways, especially the growth and vascular-related signaling pathways. In addition, given the pervasive evidence for mutual interaction between endometrial cells, this study deepened the understanding of endometrial repair and regeneration. Taken together, CellChat analysis faithfully revealed many signaling events with well-established roles in the repair and regeneration of the endometrium.

## Data Availability

The raw sequence data reported in this paper have been deposited in the Genome Sequence Archive in National Genomics Data Center (CNCB-NGDC Members and Partners, 2021), China National Center for Bioinformation/Beijing Institute of Genomics, Chinese Academy of Sciences, under accession number HRA002555 that are publicly accessible at https://bigd.big.ac.cn/gsa.

## Supplementary Material

Supplementary Sheet S1 Cellchat signaling pathways of endometrial cells.

Supplementary Sheet S2 Cellchat ligand-receptor pairs of endometrial cells.

Supplementary Sheet S3 Cellchat net count of endometrial cells.

Supplementary Sheet S4 Cellchat net weight of endometrial cells.
